# The association between the presence of fast-food outlets and BMI: the role of neighbourhood socio-economic status, healthy food outlets, and dietary factors

**DOI:** 10.1186/s12889-022-13826-1

**Published:** 2022-07-27

**Authors:** Carel-Peter L. van Erpecum, Sander K. R. van Zon, Ute Bültmann, Nynke Smidt

**Affiliations:** 1 Department of Epidemiology, University of Groningen, University Medical Center Groningen, Hanzeplein 1, 9700 RB Groningen, the Netherlands; 2 Department of Health Sciences, Community and Occupational Medicine, University of Groningen, University Medical Center Groningen, Hanzeplein 1, 9700 RB Groningen, the Netherlands

**Keywords:** Built environment, Fast foods, Body Mass Index, Overweight, Waist-Height ratio, Socioeconomic factors

## Abstract

**Background:**

Evidence on the association between the presence of fast-food outlets and Body Mass Index (BMI) is inconsistent. Furthermore, mechanisms underlying the fast-food outlet presence-BMI association are understudied. We investigated the association between the number of fast-food outlets being present and objectively measured BMI. Moreover, we investigated to what extent this association was moderated by neighbourhood socio-economic status (NSES) and healthy food outlets. Additionally, we investigated mediation by frequency of fast-food consumption and amount of fat intake.

**Methods:**

In this cross-sectional study, we used baseline data of adults in Lifelines (*N* = *149,617*). Geo-coded residential addresses were linked to fast-food and healthy food outlet locations. We computed the number of fast-food and healthy food outlets within 1 kilometre (km) of participants’ residential addresses (each categorised into null, one, or at least two). Participants underwent objective BMI measurements. We linked data to Statistics Netherlands to compute NSES. Frequency of fast-food consumption and amount of fat intake were measured through questionnaires in Lifelines. Multivariable multilevel linear regression analyses were performed to investigate associations between fast-food outlet presence and BMI, adjusting for individual and environmental potential confounders. When exposure-moderator interactions had *p*-value < 0.10 or improved model fit (∆AIC ≥ 2), we conducted stratified analyses. We used causal mediation methods to assess mediation.

**Results:**

Participants with one fast-food outlet within 1 km had a higher BMI than participants with no fast-food outlet within 1 km (B = 0.11, 95% CI: 0.01, 0.21). Effect sizes for at least two fast-food outlets were larger in low NSES areas (B = 0.29, 95% CI: 0.01, 0.57), and especially in low NSES areas where at least two healthy food outlets within 1 km were available (B = 0.75, 95% CI: 0.19, 1.31). Amount of fat intake, but not frequency of fast-food consumption, explained this association for 3.1%.

**Conclusions:**

Participants living in low SES neighbourhoods with at least two fast-food outlets within 1 km of their residential address had a higher BMI than their peers with no fast-food outlets within 1 km. Among these participants, healthy food outlets did not buffer the potentially unhealthy impact of fast-food outlets. Amount of fat intake partly explained this association. This study highlights neighbourhood socio-economic inequalities regarding fast-food outlets and BMI.

**Supplementary Information:**

The online version contains supplementary material available at 10.1186/s12889-022-13826-1.

## Introduction

Overweight and obesity are risk factors for various chronic diseases, such as cardiovascular diseases, diabetes mellitus type II, dementia, and cancer [1]. Worldwide, the adult prevalence of overweight and obesity has tripled from 13% in 1975 to 39% in 2016 [2], currently affecting over 2 billion adults [2] and contributing globally to a rise in healthcare costs [3].

Researchers have focused increasing attention on environmental determinants of overweight and obesity, and particularly on the presence of fast-food outlets [4]. Frequency of fast-food consumption is known to be associated with caloric intake and Body Mass Index (BMI) [5], but evidence regarding the association between the presence of fast-food outlets and BMI remains inconsistent [6]. This heterogeneity in results may be explained partly by the accuracy of measurement of fast-food outlet presence [7] and BMI [8]. An accurate measurement of the presence of fast-food outlets around residential addresses requires complex linkages between fast-food outlet locations and participant residential addresses. Regarding BMI, many studies in the field relied on self-reported rather than objectively measured BMI. This can result in biased associations, as individuals with a higher BMI are more likely to underreport their BMI [9].

Furthermore, the complex interplay between the presence of fast-food outlets and other environmental factors on BMI is poorly understood. While previous studies typically studied fast-food outlet presence in isolation in relation to BMI, it is increasingly recognised that the role of fast-food outlet presence may be dependent on the wider context within a complex system consisting of other built environmental and socio-economic factors [10]. Besides, knowledge on the settings in which the association between fast-food outlet presence and BMI is stronger may be used by policy makers to create healthier living environments using tailored approaches. To date, little is known about the potentially moderating role of neighbourhood socio-economic status and the presence of healthy food outlets in the association between fast-food outlet presence and BMI. Algren et al. [11] suggested that adopting a healthy lifestyle in neighbourhoods with low socio-economic status (NSES) might be more difficult because of lower social support and higher presence of neighbourhood stressors (e.g., criminality). Previous studies reported a higher number of fast-food outlets in low NSES areas [12, 13] and that the association between the presence of fast-food outlets and BMI is stronger in people from low *individual* socio-economic status [14, 15]. Still, to our best knowledge, no study has assessed whether the association between the presence of fast-food outlets and BMI is moderated by *neighbourhood* socio-economic status. Contrary to the potentially amplifying role of low NSES ubiquitous presence of healthy food outlets (e.g., fruit and vegetable markets, supermarkets [16–19]) may provide a *buffer* against the potentially unhealthy influence of fast-food outlets by enabling individuals to opt for healthy alternatives. Yet, no study has assessed whether the association between the presence of fast-food outlets and BMI is *weaker* with a higher presence of healthy food outlets.

Moreover, more research is needed on mediating pathways in the association between the presence of fast-food outlets and BMI. Elucidating such mediating pathways may provide insight into the mechanisms through which fast-food outlets may exert their health effects, and inform approaches to limit the impact of fast-food outlets. The frequency of fast-food consumption or amount of fat intake may mediate the association between the presence of fast-food outlets and BMI [20]. Frequency of fast-food consumption may have a mediating role, as the presence of fast-food outlets may promote social norms regarding eating fast-food [20]. A European cross-sectional study by Mackenbach et al. [21] found that associations between the presence of fast-food outlets and BMI were not mediated by the frequency of fast-food consumption. However, mediated associations may have been underestimated, as Mackenbach et al. had to rely on self-reported BMI. Furthermore, the amount of fat intake may mediate the association between the presence of fast-food outlets and BMI, as large amounts of fat are a major unhealthy component of fast-food meals [22]. To understand the mechanisms underlying the association between the presence of fast-food outlets and BMI, more research is needed regarding the mediating role of the frequency of fast-food consumption and the amount of fat intake.

In this study, we investigated the association between the presence of fast-food outlets and objectively measured BMI. Moreover, we investigated the moderating role of NSES and the presence of healthy food outlets in the association between the presence of fast-food outlets and objectively measured BMI. Furthermore, we examined to what extent this association is mediated by the frequency of fast-food consumption and the amount of fat intake.

## Methods

### Study population

In this cross-sectional study, we used baseline adult data (≥ 18 years) from the Lifelines Cohort Study [23]. Lifelines is a prospective population-based cohort study examining in a unique three-generational design the health and health-related behaviours of 167,729 persons living in the north of the Netherlands. It employs a broad range of investigative procedures in assessing the biomedical, socio-demographic, behavioural, physical and psychological factors, which contribute to the health and disease of the general population, with a special focus on multi-morbidity and complex genetics. Recruitment took place between December 2006 and December 2013 through general practitioners, participants’ family members, and online registrations. At baseline, participants underwent a physical examination and filled out online questionnaires. Lifelines is broadly representative of the general population of the Northern Netherlands in terms of socio-economic and lifestyle factors, prevalence of chronic diseases, and general health [24]. Participants’ residential addresses were geo-coded based on a nationwide address registry [25]. Specifically for this study, we excluded nursing home residents (*N* = *181*), as they may not be able to interact with their fast-food environment, and pregnant women (currently or last year; *N* = *2,757*), whose current BMI may not represent their actual weight status.

### Data linkage

Based on the geo-coded residential addresses of Lifelines participants, we established a linkage with LISA data (‘Landelijk Informatiesysteem van Arbeidsplaatsen’; www.lisa.nl) [26]. LISA data consist of Dutch retail outlet locations where paid work is performed for at least one hour/month. We used LISA data from 2012, corresponding with the median recruitment year of Lifelines’ baseline participants (2012).

To retrieve locations of fast-food outlets, healthy food outlets, and physical activity facilities we used specific Standard Business Information (SBI) codes. We defined fast-food outlets as outlets offering food that was (1) paid for at the counter, (2) predominantly highly caloric, unhealthy, and prepared in bulk and kept hot, and (3) meant to be eaten directly [27]. SBI-codes used to select fast-food outlets were based on a previous article on this topic [18]. We defined healthy food outlets as outlets offering food that was (1) predominantly unprocessed and meant to be prepared at home and (2) predominantly healthy. In line with existing literature [16, 18] and given the evidence that fruits, vegetables, and fatty fish are associated with a healthier cardiometabolic profile [28], we included the following retail outlets as healthy food outlets: retail outlets for potatoes, vegetables, and fruit (SBI code 47.21); retail outlets for natural foods and reform articles (e.g., Ekoplaza) (SBI code 47.29.2); marketplace for potatoes, vegetables, and fruit (SBI code 47.81.1); retail outlets for selling fish (SBI code 47.23); supermarkets and similar retail outlets with a general assortment of foods (SBI code 47.11). Supermarkets are also generally considered a source of healthy foods in the literature [16], even though they also offer unhealthy foods. We defined physical activity facilities as facilities that (1) require an access fee and (2) are meant exclusively for individuals to exercise. Further details about the definitions are provided elsewhere [29] and in Additional file 1: Table S1.

Additionally, we linked participants’ neighbourhood codes to Statistics Netherlands 2012 neighbourhood data [30]. We determined neighbourhood boundaries using official administrative definitions from Statistics Netherlands [31]. Based on these boundaries, a neighbourhood in the Netherlands covered a median (IQR) surface of 84 (35–289) hectares and contained a median (IQR) of 660 (180–1,850) residents.

### Exposure

Based on the linkage with LISA data, we computed the number of fast-food outlets within a straight-line 1-kilometre (km) distance of participants’ residential addresses. We opted for an absolute rather than a relative measure of fast-food outlet presence, as relative measures are more difficult to interpret and to translate into policies on the food environment [32]. The 1 km-density was based on a previous study in the Dutch context that found strongest associations with BMI for 1 km-density, rather than proximity and density of other ranges [29]. Moreover, the distance of 1 km is equal to a 10- to 15-minute walk for an average adult, and has been linked to food shopping behaviours [33]. We did not observe a linear relation between the number of fast-food outlets within 1 km and BMI in our data. Therefore, we categorised the number of fast-food outlets into null, one, or at least two fast-food outlets within 1 km. This categorisation reflects being present versus being absent, and allows for further assessment when multiple fast-food outlets are present within 1 km. Splitting the group of participants with at least two fast-food outlets within 1 km into more categories was not opted for, as a study on the Dutch context found that going from null to one fast-food outlet was associated with changes in BMI, but that an increase in fast-food outlets was not associated with changes in BMI when multiple fast-food outlets in the environment were available already [13]. We computed this variable using address points in QGIS v3.4.2 (match rate 99.6%).

### Outcomes

BMI (in kilogram(kg)/metre(m)^2^) was based on objectively measured weight (in kg) and height (in centimetre(cm)), without shoes and heavy clothing, during a physical examination at the Lifelines research study site.

### Moderators

We determined neighbourhood socio-economic status (NSES) based on the linkage with Statistics Netherlands, and measured it as a composite score of: (1) the average value of a house per 1,000 euros, (2) the percentage of owner-occupied houses, (3) the mean net disposable monthly income, and (4) the percentage of individuals aged 15–65 years receiving assistance benefits. We opted for these indicators to create a multidimensional NSES score, reflecting the financial, housing, and work situation in a neighbourhood [34]. After reversing the fourth indicator, we aggregated all indicators into one z-standardized score using principal component analysis. Loadings of the separate indicators on the NSES variable were all 0.80 or higher. The NSES variable was z-standardised and divided into tertiles (low, middle and high NSES).

The number of healthy food outlets within 1 km of the residential address was based on the linkage with LISA data and categorized into null, one and at least two healthy food outlets within 1 km of the residential address.

### Mediators

The frequency of fast-food consumption was measured with the question: ‘If you have eaten ready-to-eat meals in the past month, how often did you eat meals from fast-food restaurants (e.g. McDonalds, Burger King or KFC)?’ with the following answer options: ‘never’, ‘sometimes’, ‘often’, and ‘always’. Since only a relatively small proportion of participants responded with ‘often’ (4.1%) or ‘always’ (1.8%), we merged these two categories with the response option ‘sometimes’. The amount of fat intake was based on a 110-item Food Frequency Questionnaire (FFQ) [28] to assess past-month food intake, and on a Dutch food composition database [35]. We measured the amount of fat intake as the number of grams of fat/1,000 kcal, as FFQ’s can be used to accurately estimate only *relative* intakes of certain foods over other types of foods [28]. Further details about the measurement of fat intake in Lifelines are provided elsewhere [28, 36].

### Potential confounders

Analyses were adjusted for individual-level and neighbourhood-level potential confounders. Individual-level potential confounders included: sex; age (in years); partner status (having a partner or not); highest level of completed education (low [less than primary education, primary education, or lower secondary education], middle [upper secondary education or post-secondary non-tertiary education], and high [short-cycle tertiary education, bachelor or equivalent education, master or equivalent education, doctoral or equivalent education] based on the International Standard Classification of Education [37]); weekly working hours (0, 1–11, 12–19, 20–31, or ≥ 32 h); net monthly income (middle value of categories <€750 [set to €500], €750-€1,000, subsequent 500-euro intervals until €3,500, and >€3,500 [set to €3,750], divided by the square root of individuals living from that income [38]); density of physical activity facilities within 1 km (based on the LISA data); household size (number of individuals living in the household); density of healthy food outlets within 1 km; and occupational prestige (based on the Standard International Occupational Prestige Scale (SIOPS) [39]). Neighbourhood-level potential confounders were address density (number of addresses/km^2^) and NSES, as based on the linkage with Statistics Netherlands. For mediation analyses, we additionally included (1) occupational and (2) non-occupational moderate-to-vigorous physical activity (MVPA) based on the Short Questionnaire to Assess Health-enhancing physical activity (SQUASH) questionnaire [40], as physical activity may influence diet and BMI, but is unlikely to influence the presence of fast-food outlets. Occupational and non-occupational MVPA were treated separately as they are differentially associated with BMI [41].

### Statistical analysis

We imputed missing data using Multiple Imputation by Chained Equations with Multilevel Data (MICEMD), accounting for clustered data within neighbourhoods. Based on statistical recommendations [42], we created 10 imputed datasets.

To investigate associations between the presence of fast-food outlets and BMI, we performed multivariable multilevel linear regression analyses to account for clustered data within neighbourhoods. We adjusted for the potential confounders listed above, and reported effect sizes and 95% confidence intervals. To examine moderation by NSES and healthy food outlets, we assessed their two-way interaction terms with fast-food outlet presence on BMI and tested the model fit (Akaike Information Criterion (AIC)) for models with and without these interaction terms. If one of the interaction terms had a *p*-value < 0.10 [43]) or model fit improved meaningfully (i.e., at least 2 points lower AIC in the model with interaction terms [44]), we presented stratified analyses for NSES and/or healthy food outlet presence. In addition, we reasoned that the association between fast-food outlet presence and BMI may be especially pronounced in low NSES areas with few healthy food outlets. Therefore, we tested three-way interaction terms between fast-food outlet presence, NSES, and healthy food outlet presence, and tested the AIC for models with and without these interaction terms. If one of the three-way interaction terms had a *p*-value < 0.10 or model fit improved meaningfully (i.e., at least 2 points lower AIC in the model with interaction terms), we presented stratified analyses by level of NSES and number of healthy food outlets.

To examine mediation through the frequency of fast-food consumption and the amount of fat intake, we performed mediation analyses using causal mediation methods [45]. Multilevel models were used to estimate paths. We incorporated interactions between exposure and mediators [46], as associations between the presence of fast-food outlets and BMI may be weaker among individuals who never consume fast-food [47] or fatty foods. In the mediation analyses we adjusted for all potential confounders of the main analyses. In the mediator-outcome paths we additionally adjusted for (1) occupational and (2) non-occupational MVPA.

To evaluate the robustness of the results we conducted three sensitivity analyses. First, we repeated the analyses with waist-to-height ratio as the outcome. Although BMI is a common weight status outcome in the literature and is quick and easy to assess, BMI-based measures are criticised for not adequately reflecting fat mass and regional fat distribution [48]. As a result, using BMI may introduce the risk of misclassifying participants as being overweight or obese, while the elevated BMI is due to, for instance, higher muscle mass. Waist-to-height ratio more accurately reflects fat mass [49] and central adiposity, and is less susceptible to misclassification than BMI [50]. Also, waist-to-height ratio more accurately predicts chronic disease occurrence [51] and all-cause mortality [48]. Second, we repeated the analysis with only those participants recruited after January 1st, 2012 (*N* = *79,697*), to examine the potential influence of a temporal mismatch between the measurement of exposure (2012) and outcome (2006–2013). Third, we repeated the mediation analysis by taking the frequency of fast-food consumption categorically instead of dichotomously. This allowed us to distinguish between individuals that ‘never’, ‘sometimes’, ‘often’, or ‘always’ consume fast-food.

## Results

We included 149,617 participants from 3,509 neighbourhoods. The mean (sd) BMI was 26.1 (4.3) kg/m^2^. The mean (sd) age was 44.8 (13.1), and 57.7% of the participants were female. The percentages of participants with null, one or at least two fast-food outlets within 1 km of the residential address were 22.3%, 13.4%, and 64.2%, respectively (Table 1).

**Table 1:** Characteristics of study population

Variable	Total study population (*N* = 149,617)	0 fast-food outlets within 1 km (*N* = *33,407*)	1 fast-food outlet within 1 km (*N* = *20,055*)	≥ 2 fast-food outlets within 1 km (*N* = *96,054*)
Age (in years), mean (sd)	44.8 (13.1)	45.4 (11.9)	45.1 (12.3)	44.6 (13.6)
Sex				
Female, *N* (%)	*86,382* (57.7)	*19,078* (57.1)	*11,395* (56.8)	*55,848* (58.1)
Partner status				
Having a partner, *N* (%)	*124,696* (85.3)	*29,459* (90.1)	*17,526* (89.3)	*77,639* (82.8)
Education				
Low, *N* (%)	*42,644* (29.9)	*9,243* (28.7)	*6,124* (31.8)	*27,248* (29.9)
Middle, *N* (%)	*57,192* (40.1)	*13,361* (41.5)	*7,962* (41.3)	*35,839* (39.3)
High, *N* (%)	*42,845* (30.0)	*9,587* (29.8)	*5,177* (26.9)	*28,055* (30.8)
Income, net euros per month, mean (sd)	1,532 (581)	1,548 (575)	1,529 (559)	1,527 (587)
Occupational prestige score, median (IQR)	43.3 (26.6–65.0)	42.3 (26.0–65.0)	41.3 (26.6–61.8)	43.9 (26.6–65.0)
Weekly working hours				
0 (not working), *N* (%)	*4,712* (33.3)	*10,283* (31.8)	*6,107* (31.6)	*31,367* (34.2)
1–11 h, *N* (%)	*4,839* (3.4)	*1,224* (3.8)	*741* (3.8)	*2,872* (3.1)
12–19, *N* (%)	*9,496* (6.6)	*2,354* (7.3)	*1,460* (7.5)	*5,678* (6.2)
20–31 h, *N* (%)	*25,705* (17.9)	*6,017* (18.6)	*3,629* (18.7)	*16,049* (17.5)
≥32 h, *N* (%)	*55,782* (38.8)	*12,493* (38.6)	*7,418* (38.3)	*35,858* (39.1)
Household size (total number of members in household), median (IQR)	3 (2–4)	3 (2–4)	3 (2–4)	3 (2–4)
Frequency of fast-food consumption				
Never, *N* (%)	*57,817* (57.8)	*12,664* (58.9)	*7,510* (57.3)	*37,618* (57.6)
Sometimes, *N* (%)	*36,272* (36.3)	*7,608* (35.4)	*4,797* (36.6)	*23,852* (36.5)
Often, *N* (%)	*4,058* (4.1)	*848* (3.9)	*546* (4.2)	*2,663* (4.1)
Always, *N* (%)	*1,808* (1.8)	*394* (1.8)	*258* (2.0)	*1,156* (1.8)
Amount of fat intake, g/1000 kcal, mean (sd)	38.5 (7.2)	38.6 (7.5)	38.7 (7.1)	38.5 (7.1)
Physical activity				
Occupational moderate-to-vigorous physical activity, in minutes per week, median (IQR)	0 (0–30)	0 (0–66)	0 (0–79)	0 (0–0)
Non-occupational moderate-to-vigorous physical activity, in minutes per week, median (IQR)	210 (60–450)	180 (30–420)	180 (45–420)	230 (70–460)
Body Mass Index (in kg/m^2^), mean (sd)	26.1 (4.3)	26.0 (4.2)	26.2 (4.3)	26.1 (4.4)
Overweight (BMI 25.0–29.9), *N* (%)	*58,500* (36.9)	*13,347* (40.0)	*8,080* (40.3)	*37,037* (38.6)
Obesity (BMI ≥ 30.0), *N* (%)	*23,395* (14.8)	*4,862* (14.6)	*3,249* (16.2)	*15,272* (15.9)
Waist circumference in cm, mean (sd)	90.2 (12.5)	90.2 (12.1)	90.8 (12.4)	90.1 (12.7)
Elevated waist circumference (≥ 88 cm for women or ≥ 102 cm for men)	*52,344* (35.0)	*11,313* (33.9)	*7,196* (35.9)	*33,798* (35.2)
Waist-height ratio, mean (sd)	0.52 (0.07)	0.51 (0.07)	0.52 (0.07)	0.52 (0.07)
Number of fast-food outlets within 1 km, median (IQR)	3 (1–8)	0 (0–0)	1 (1–1)	6 (3–12)
Neighbourhood address density (addresses/km^2^), median (IQR)	616 (208–1,155)	103 (50–292)	254 (144–573)	939 (509–1,485)
Neighbourhood socio-economic status (NSES), mean (sd)^a^	-0.01 (1.01)	0.62 (0.73)	0.25 (0.83)	-0.27 (1.00)
Number of healthy food outlets within 1 km, median (IQR)	2 (1–5)	0 (0–1)	1 (0–1)	4 (2–6)
Number of physical activity facilities within 1 km, median (IQR)	1 (0–3)	0 (0–1)	1 (0–2)	2 (1–4)

*IQR* Interquartile range, *sd* Standard deviation; Note: Characteristics are based on non-imputed data. For categorical variables, percentages per category represent valid percentages. ^a^ Neighbourhood socio-economic status is a composite score based on (1) the average value of a house per 1,000 euros, (2) the percentage of owner-occupied houses, (3) the mean net disposable monthly income, and (4) the percentage of individuals aged 15–65 years receiving assistance benefits, with a higher score indicating a higher neighbourhood socio-economic status

### Association between the presence of fast-food outlets and body Mass Index

Participants who had one fast-food outlet within 1 km of their residential address had a higher BMI than participants with no fast-food outlets within a 1 km radius (BMI: B = 0.11, 95% CI: 0.01, 0.21; Table 2). Participants with two or more fast-food outlets within 1 km did not have a higher BMI than participants with no fast-food outlets within the same radius (BMI: B = 0.10, 95% CI: -0.01, 0.20; Table 2).

**Table 2:** The association between fast-food outlet presence and Body Mass Index

Number of fast-food outlets within 1 km	*N* (%)	Unadjusted model, B (95% CI)	Adjusted model, B (95% CI)^a^
0 fast-food outlets	*33,407* (22.3)	ref	ref
1 fast-food outlet	*20,055* (13.4)	**0.17 (0.07, 0.27)**	**0.11 (0.01, 0.21)**
≥ 2 fast-food outlets	*96,054* (64.2)	0.05 (-0.03, 0.13)	0.10 (-0.01, 0.20)

^a^: analyses were adjusted for age, sex, partner status, highest level of completed education, weekly working hours, income, number of physical activity facilities within 1 km, household size, number of healthy food outlets within 1 km, neighbourhood socio-economic status, occupational prestige, and address density. Note: Bold values represent associations with *p* < 0.05

The *p*-value of interaction terms were 0.06 for fast-food outlet presence and NSES (two-way interaction), 0.16 for fast-food outlet presence and healthy food outlet presence (two-way interaction), and 0.01 for fast-food outlet presence, NSES, and healthy food outlet presence (three-way interaction). None of the models with interaction terms had a better fit than models without interaction terms based on the AIC.

### Association between the presence of fast-food outlets and Body Mass Index, stratified by neighbourhood socioeconomic status (NSES)

**Fig. 1:**
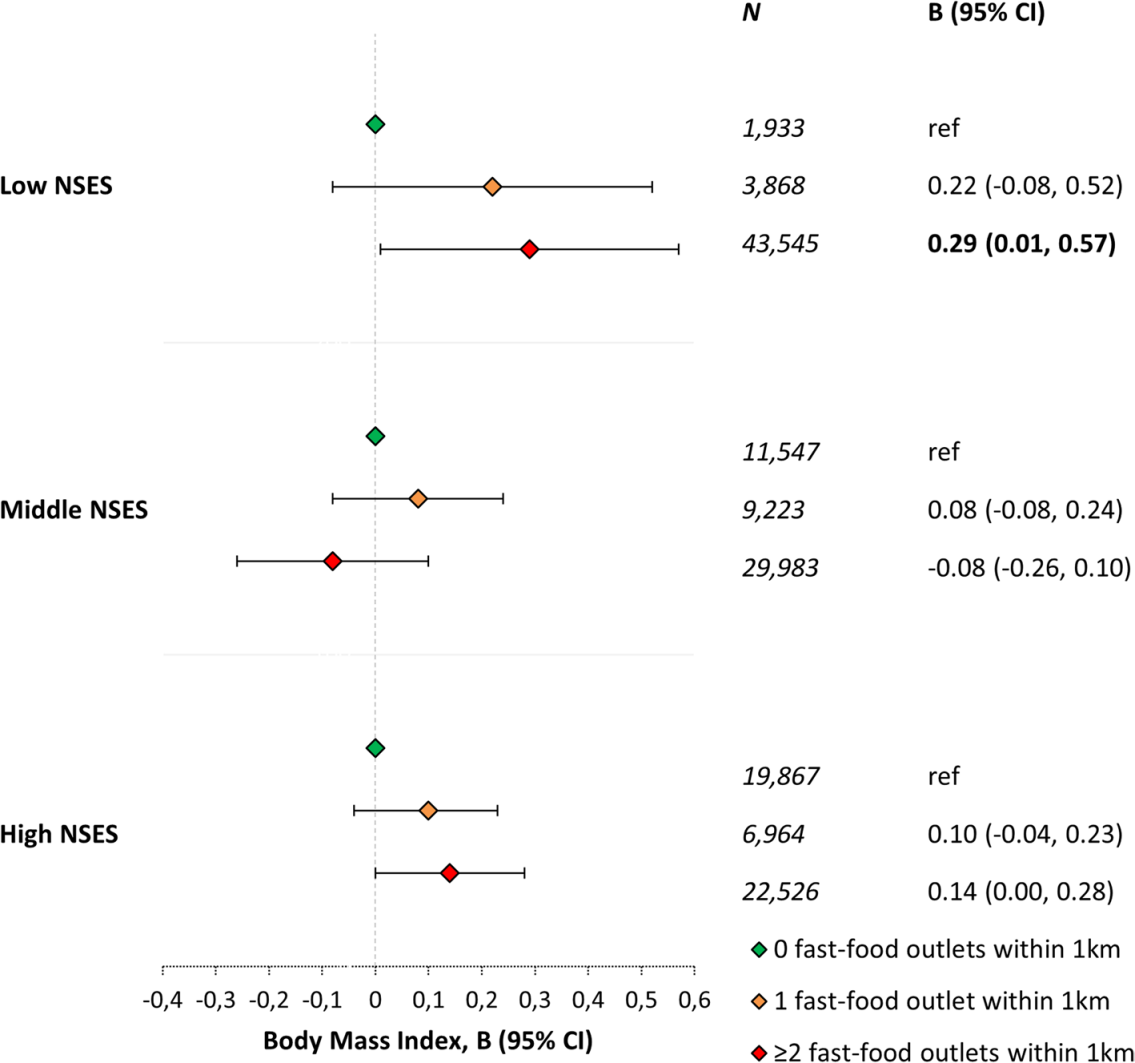
Associations between the presence of fast-food outlets and Body Mass Index, stratified for participants living in neighbourhoods with low, middle, and high socio-economic status (based on: (1) average value of a house per 1,000 euros; (2) percentage of owner-occupied houses; (3) mean net disposable monthly income; and (4) percentage of individuals aged 15–65 years receiving assistance benefits). Associations were adjusted for age, sex, partner status, highest level of completed education, weekly working hours, income, number of physical activity facilities within 1 km, household size, number of healthy food outlets within 1 km, occupational prestige, and address density. Note: bold numbers represent associations with *p* < 0.05

The median (interquartile range (IQR)) number of fast-food outlets within 1 km in neighbourhoods of low, middle, and high SES were 7 (3–17), 2 (1–5), and 1 (0–4), respectively (Additional file 1: Table S2). The mean (sd) BMI of participants living in neighbourhoods of low, middle, and high SES was 26.3 (4.7), 26.1 (4.3), and 25.8 (4.1), respectively. Participants from neighbourhoods with low SES also had a lower income and reported more often a low educational level than participants from neighbourhoods with middle and high SES (Additional file 1: Table S2). Participants living in low SES neighbourhoods with at least two fast-food outlets within 1 km of their residential address had a higher BMI (BMI: B = 0.29, 95% CI: 0.01, 0.57; Fig. 1) than participants in low SES neighbourhoods with no fast-food outlet within 1 km of their residential address. The density of fast-food outlets was not associated with BMI among participants with middle NSES. In high SES neighbourhoods, fast-food outlet density within 1 km was borderline associated with BMI, with weaker effect sizes than in low SES neighbourhoods (B = 0.14, 95% CI: 0.00, 0.28; *p* = 0.07; Fig. 1).

### Association between the presence of fast-food outlets and Body Mass Index, stratified by neighbourhood socioeconomic status (NSES) and the presence of healthy food outlets

**Fig. 2:**
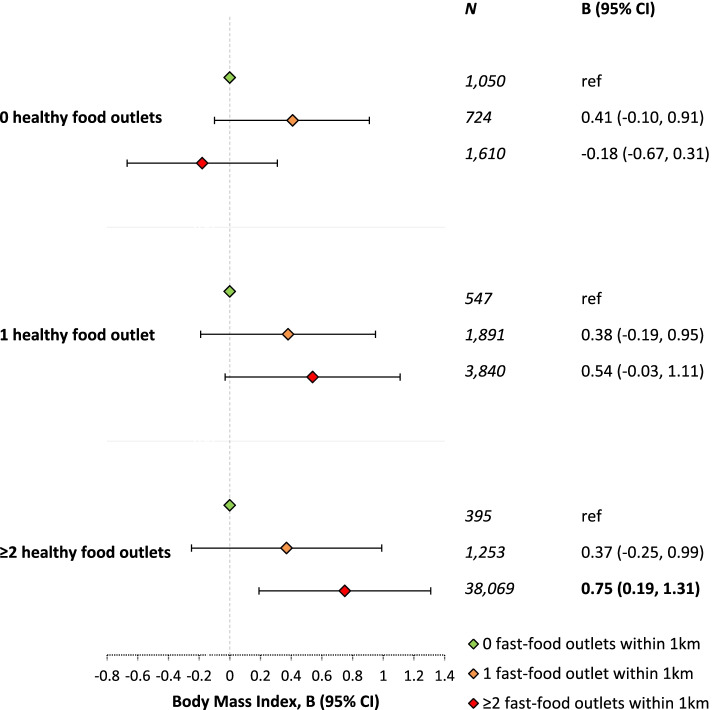
Association between the presence of fast-food outlets and Body Mass Index for participants living in low SES neighbourhoods, stratified according to number of healthy food outlets within 1 km. Associations were adjusted for age, sex, partner status, highest level of completed education, weekly working hours, income, number of physical activity facilities within 1 km, household size, occupational prestige, and address density. Note: bold numbers represent associations with *p* < 0.05

For participants with low NSES, the association between the presence of fast-food outlets and BMI was not attenuated by the availability of healthy food outlets within 1 km of their residential address (Fig. 2). To the contrary, the association between the presence of fast-food outlets and BMI among participants with low NSES was more pronounced if at least two healthy food outlets were available within 1 km of their residential address (B = 0.75, 95% CI: 0.19, 1.31; Fig. 2). For participants with middle or high NSES, a clear moderation pattern by the presence of healthy food outlets within 1 km was lacking (Additional file 1: Table S5).

### Mediation through frequency of fast-food consumption and amount of fat intake

**Fig. 3:**
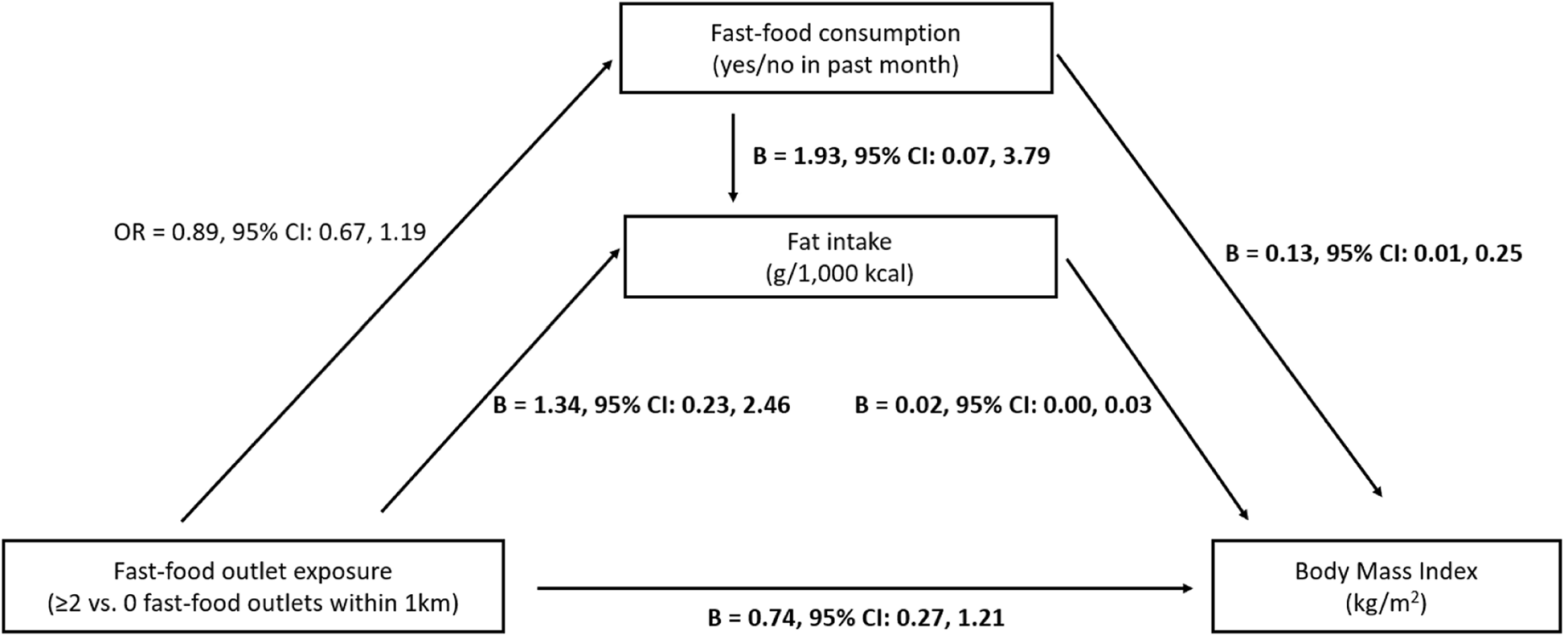
Results of causal mediation analyses to investigate mediation through frequency of fast-food consumption and amount of fat intake in the association between the presence of fast-food outlets and Body Mass Index, in a subgroup of participants living in low SES neighbourhoods with at least two healthy food outlets within 1 km (*N* = *39,717*). Associations were adjusted for age, sex, partner status, highest level of completed education, weekly working hours, income, number of physical activity facilities within 1 km, household size, number of healthy food outlets within 1 km, neighbourhood socio-economic status, occupational prestige, and address density. In the mediator-outcome associations, we also adjusted for occupational and non-occupational moderate-to-vigorous physical activity. Note: bold numbers represent associations with *p* < 0.05

Among participants with low NSES with at least two healthy food outlets within 1 km, associations between the presence of fast-food outlets and BMI were partly (for 3.1%) explained by the amount of fat intake, but not by the frequency of fast-food consumption (Fig. 3 and Additional file 1: Table S3). These participants who lived with at least two fast-food outlets within 1 km had a higher fat intake than those who had no fast-food outlet within 1 km (B = 1.34 g/1,000 kcal, 95% CI: 0.27, 2.42). Subsequently, higher fat intake was associated with a higher BMI (per g/1,000 kcal: B = 0.02, 95% CI: 0.01, 0.02).

### Sensitivity analyses

Repeating the analyses with waist-to-height ratio as outcome did not affect the conclusions (Additional file 1: Table S6-S8). Furthermore, repeating the analysis with only the 79,697 participants recruited from January 1st, 2012 did not affect the conclusions (Additional file 1: Table S9-S10). Also, the findings of the mediation analysis were not affected by taking the frequency of fast-food consumption categorically instead of dichotomously (Additional file 1: Table S11).

## Discussion

This study indicates that individuals living in low SES neighbourhoods with at least two fast-food outlets within 1 km of their residential address have a higher BMI than individuals with no fast-food outlet within the same radius. Rather than being attenuated by the presence of healthy food outlets, these associations were only more pronounced. The amount of fat intake, but not the frequency of fast-food consumption, explained only a limited part of the association between the presence of fast-food outlets and BMI.

The current study emphasizes the role of fast-food outlets in inequalities between individuals living in neighbourhoods with a different socioeconomic status. First, the number of fast-food outlets itself was substantially higher in low SES neighbourhoods than in high SES neighbourhoods: the median (IQR) number of fast-food outlets within 1 km for participants with low, middle, or high NSES was 7 (3–17), 2 (1–5), and 1 (0–4), respectively. This is in line with several other studies that found more fast-food outlets to be present in low NSES areas [52]. Second, the mean BMI was higher among participants from low NSES areas (26.3 kg/m^2^) than among participants with middle (26.1 kg/m^2^) or high NSES (25.8 kg/m^2^). Third, although we also saw a borderline significant association between fast-food outlet presence and BMI in high NSES areas, the strongest effect sizes of fast-food outlet presence on BMI were observed in low NSES areas. This may be because low SES neighbourhoods have less social capital [53] and more positive social norms that encourage eating fast-food [54]. In low SES neighbourhoods, we found effect sizes up to 0.75 of fast-food outlet presence on BMI, averaging a higher weight of 2.30 kg for a Dutch adult of average height (i.e., an average of 1.75 metre (m) for males and females combined [55]). By examining the role of SES on neighbourhood level, this study may provide a tangible platform for conducting public health interventions and contribute to a better reach of such interventions towards vulnerable groups [56].

Remarkably, associations between the presence of fast-food outlets and BMI in low SES neighbourhoods were more pronounced with increasing availability of healthy food outlets (at least two). This may suggest that healthy food outlets do not buffer the potentially unhealthy impact of fast-food outlets. On the one hand, this finding may be the result of our inclusion of supermarkets in the definition of healthy food outlets. Although in the literature supermarkets are typically considered as healthy food outlets [16–19], they also offer a wide range of unhealthy foods [57]. Even more, a study found that 71% of all promoted food products in supermarkets in the Netherlands do not contribute to a healthy diet [58]. On the other hand, this finding may be explained by *self-licensing* dietary behaviours [59]: healthy food outlet shopping could be used as a psychological ‘license’ to consume fast-food. Also, low SES neighbourhoods with many healthy food outlets may at the same time be urbanised areas with easy access to all types of foods. In such areas, snacking patterns may be more common [60].

Associations between the presence of fast-food outlets and BMI were only modestly explained by the amount of fat intake. The high amounts of fat in fast-food meals [22] may explain these observed mediation effects. The associations were not explained by the frequency of fast-food consumption. A reason for this could be that only a small proportion (5.9%) of individuals indicated that they ‘often’ or ‘always’ consumed fast-food. Furthermore, we did not take into account the *amount* of fast-food consumed. We can also not rule out differential misclassification, as the frequency of fast-food consumption was based on self-report: individuals with a higher BMI may have given socially desirable answers, and underreported their frequency of fast-food consumption [60].

Strengths of this study include its use of objectively measured BMI, its large sample size, and the overall representativeness of the study sample [24]. Moreover, the study sample came from a large rather than narrow geographical area (e.g., a city). However, this study also has limitations. Firstly, as the results are based on cross-sectional data, we cannot exclude the possibility of reverse causation. For instance, fast-food outlets may have opened selectively in areas where BMI is higher or where fast-food meals or fatty foods are consumed more often. Therefore, we cannot draw firm conclusions about the causal relationship between the presence of fast-food outlets and BMI and underlying causal mechanisms. Longitudinal studies, and particularly natural experiments, are needed to strengthen the evidence regarding the impact of the presence of fast-food outlets on BMI. Secondly, we cannot rule out the effect of fast-food delivery services, even though before 2014 the use of these services was less common than today [52]. This may have led to underestimated associations in the current study. Thirdly, we relied on straight-line distances instead of street-network distances to compute the presence of fast-food outlets. Even though there is evidence that straight-line and street-network distances correlate highly [61], this correlation may have been weaker in rural areas where a part of the Lifelines participants reside. Fourthly, even though we adjusted for address density in our analyses, we cannot rule out that results from this observational study are affected by address density due to residual confounding. In urban areas, fast-food outlets are more ubiquitous, while the average BMI is lower [29].

This study provides a deeper understanding of the role of the presence of fast-food outlets in BMI, *who* might be most affected, and *how* the presence of fast-food outlets may influence BMI. We hope that our results can be used by policy-makers to create healthier food environments. In the future, longitudinal studies are needed to strengthen the evidence on how the presence of fast-food outlets affects changes in BMI over time. Moreover, future studies should examine overweight and obesity using systems dynamics approaches. Such approaches model overweight and obesity as part of a complex system [62–64], and could thereby move beyond bivariate exposure-outcome associations.

## Conclusions

We found that among individuals living in low SES neighbourhoods, the presence of fast-food outlets is associated with objectively measured BMI. These associations were not buffered by the presence of healthy food outlets and only modestly explained by the amount of fat intake. Our results may provide a stepping stone toward understanding the widespread pandemic of overweight and obesity, and how the fast-food environment may contribute to health inequalities.

## Supplementary Information

**Additional file 1.**

## Data Availability

Researchers can apply for the data and biomaterial through a proposal submitted to the LifeLines Cohort Study. For more information, we refer to www.lifelines.nl.

## Abbreviations

BMI: Body Mass Index
cm: centimetre
FFQ: Food Frequency Questionnaire
IQR: Interquartile range
kg: kilogram
km: kilometre
m: metre
LISA: Landelijk Informatiesysteem van Arbeidsplaatsen
MICEMD: Multiple Imputation by Chained Equations with multilevel data
NSES: Neighbourhood socio-economic status
sd: standard deviation
SIOPS: Standard International Occupational Prestige Scale
SQUASH: Short Questionnaire to Assess Health-enhancing physical activity
