# Supplementary material for: The association between the presence of fast-food outlets and BMI: the role of neighbourhood socio-economic status, healthy food outlets, and dietary factors
Source: BMC Public Health. 2022 Jul 27;22:1432. doi: 10.1186/s12889-022-13826-1 (PMC9331587; doi:10.1186/s12889-022-13826-1)
Supplement: Supplementary file 1 — Additional file 1. [file 12889_2022_13826_MOESM1_ESM.docx]

**Supplementary Material**

**Table S1**. Standard Business Information codes used to identify fast-food outlets, healthy food outlets, BMI

neutral food outlets, and physical activity facilities.

| **Outlet type** | **SBI (Standardised Business Information) code** |
| --- | --- |
| **Fast-food outlets** |  |
| Outlets selling predominantly meat products | 47.22.1 |
| Outlets selling predominantly in bread and banquet | 47.24.1 |
| Outlets selling predominantly chocolate and confectionary | 47.24.2 |
| Outlets identified as restaurants or hotel-restaurants containing at least one of the following elements in their name: ‘pizz’, ‘ijssalon’, ‘snack’, ‘cafetaria’, ‘afhaal’, ‘shoarma’, ‘pannenk’, ‘pannek’, ‘stroop’, ‘wegr’, ‘steak’, ‘rib’, ‘snelbuffet’, ‘venez’, ‘kwalitaria’, ‘alanya’, ‘antalya’, ‘cairo’, ‘shalom’, ‘anatolya’, ‘shaam’, ‘cleopatra’, ‘sahara’, ‘hasret’, ‘ozan’, ‘kebab’, ‘pyramide’, ‘habibi’, ‘cevdet’, ‘ali baba’, ‘babylon’, ‘dönerix’, ‘shaka’, ‘istanbul’, ‘pappa joe’, ‘McDonalds’, ‘Kentucky Fried Chicken’, ‘KFC’, ‘sub’ | 55.10.1 (hotel-restaurants) and 56.10.1 (restaurants) |
| Lunch rooms, snack bars, ice cream parlours, food stalls | 56.10.2 |
| **Healthy food outlets** |  |
| Supermarkets and similar retail outlets with a general assortment of foods | 47.11 |
| Retail outlets for potatoes, vegetables, and fruit | 47.21 |
| Retail outlets for natural foods and reform articles (e.g., Ekoplaza) | 47.29.2 |
| Marketplace for potatoes, vegetables, and fruit | 47.81.1 |
| Retail outlet for selling fish | 47.23 |
| **Physical activity facilities** |  |
| Fitness centres | 93.13 |
| Sport fields | 93.11.1 |
| Sport halls | 93.11.2 |
| Gymnastics halls | 93.11.3 |
| Other physical activity facilities | 93.11.9 |
| Soccer pitches | 93.12.1 |
| Pitches for any team sport (excluding soccer) | 93.12.2 |
| Athletics tracks | 93.12.3 |
| Tennis courts | 93.12.4 |
| Facilities for horse-riding; stables | 93.12.5 |
| Bicycling sport tracks | 93.12.6 |
| Winter sport facilities | 93.12.8 |
| Other outdoor physical activity facilities | 93.12.9 |
| Indoor facilities for individual sports (e.g., climbing hall) | 93.14.1 |
| Indoor facilities for team sports (e.g., basketball) | 93.14.2 |
| Indoor facilities for sports to test physical strength and/or martial arts | 93.14.3 |
| Indoor facilities for bowling, skittles, and pool | 93.14.4 |
| Gyms | 93.14.6 |
| Other indoor physical activity and team sport facilities | 93.14.9 |
| Swimming sport halls | 93.15.1 |
| Facilities for rowing, canoeing, sailing and surfing | 93.15.2 |

**Table S2.** Characteristics of total study population (*N* = *149,617*), separately for participants with low, middle, or high neighbourhood socio-economic status (NSES)^1^.

| **Variable** | Low NSES  (*N* = *49,926*) | Middle NSES (*N* = *50,312*) | High NSES  (*N* = *49,379*) |
| --- | --- | --- | --- |
| Age (in years), mean (SD) | 43.5 (13.8) | 44.7 (12.9) | 46.2 (12.4) |
| Sex |  |  |  |
| Female, *N* (%) | *20,375* (41.3) | *28,937* (57.5) | *28,441* (57.0) |
| Partner status |  |  |  |
| Having a partner, *N* (%) | *38,136* (79.4) | *42,674* (86.7) | *43,886* (89.7) |
| Education |  |  |  |
| Low, *N* (%) | *15,631* (33.6) | *15,015* (31.1) | *11,998* (25.0) |
| Middle, *N* (%) | *18,363* (39.5) | *20,011* (41.5) | *18,818* (39.2) |
| High, *N* (%) | *12,506* (26.9) | *13,206* (27.4) | *17,133* (35.7) |
| Income, net euros per month, mean (SD) | 1,449 (579) | 1,516 (569) | 1,630 (581) |
| Occupational prestige score, median (IQR) | 43.5 (20.6) | 44.4 (20.7) | 48.3 (21.3) |
| Weekly working hours |  |  |  |
| 0 (not working), *N* (%) | *17,195* (36.7) | *15,568* (32.1) | *15,049* (31.2) |
| 1-11 hours, *N* (%) | *1,507* (3.2) | *1,743* (3.6) | *1,589* (3.3) |
| 12-19, *N* (%) | *2,858* (6.1) | *3,481* (7.2) | *3,157* (6.5) |
| 20-31 hours, *N* (%) | *7,638* (16.3) | *8,868* (18.3) | *9,199* (19.1) |
| ≥32 hours, *N* (%) | *17,623* (37.6) | *18,903* (38.9) | *19,256* (39.9) |
| Household size (total number of members in household, median (IQR) | 2 (2 – 4) | 3 (2 – 4) | 3 (2 – 4) |
| Frequency of fast-food consumption |  |  |  |
| Never, *N* (%) | *18,324* (56.2) | *19,540* (57.5) | *19,953* (59.8) |
| Sometimes, *N* (%) | *12,267* (37.6) | *12,405* (36.5) | *11,600* (34.8) |
| Often, *N* (%) | *1,450* (4.4) | *1,414* (4.2) | *1,194* (3.6) |
| Always, *N* (%) | *577* (1.8) | *598* (1.8) | *633* (1.9) |
| Amount of fat intake, g/1000 kcal, mean (SD) | 38.3 (7.3) | 38.6 (7.1) | 38.6 (7.1) |
| Physical activity |  |  |  |
| Occupational moderate-to-vigorous physical activity, in minutes per week, median (IQR) | 0 (0 – 61) | 0 (0 – 104) | 0 (0 – 0) |
| Non-occupational moderate-to-vigorous physical activity, in minutes per week, median (IQR) | 210 (60 – 450) | 200 (60 – 425) | 220 (60 – 465) |
| Body Mass Index (in kg/m^2^), mean (SD) | 26.3 (4.7) | 26.1 (4.3) | 25.8 (4.1) |
| Overweight (BMI 25.0–29.9), *N* (%) | *18,567* (37.6) | *20,136* (40.0) | *19,797* (39.7) |
| Obesity (BMI≥30.0), *N* (%) | *8,859* (17.9) | *7,892* (15.7) | *6,644* (13.3) |
| Waist circumference, in cm, mean (SD) | 90.7 (13.1) | 90.4 (12.4) | 89.6 (12.0) |
| Elevated waist circumference (≥88 cm for women or ≥102 cm for men) | *18,445* (37.4) | *17,817* (35.4) | *16,082* (32.2) |
| Waist-height ratio, mean (SD) | 0.52 (0.08) | 0.52 (0.07) | 0.51 (0.07) |
| Number of fast-food outlets within 1 km, median (IQR) | 7 (3 – 17) | 2 (1 – 5) | 1 (0 – 4) |
| 0 fast-food outlets within 1km, *N* (%) | *1,992* (4.0) | *11,380* (22.7) | *20,035* (40.1) |
| 1 fast-food outlet within 1km, *N* (%) | *3,868* (7.8) | *9,209* (18.3) | *6,978* (14.0) |
| ≥2 fast-food outlets within 1km, *N* (%) | *43,519* (88.1) | *29,622* (59.0) | *22,913* (45.9) |
| Neighbourhood address density (addresses/km^2^), median (IQR) | 1,155 (701 – 1,720) | 427 (169 – 790) | 376 (86 – 812) |
| Neighbourhood socio-economic status^1^, mean (SD) | -1.07 (0.62) | 0.02 (0.22) | 1.00 (0.58) |
| Number of healthy food outlets within 1 km, median (IQR) | 4 (2 – 8) | 2 (1 – 4) | 1 (0 – 2) |
| Number of physical activity facilities within 1km, median (IQR) | 3 (1 – 5) | 1 (0 – 3) | 1 (0 – 2) |

IQR: Interquartile range, SD: Standard deviation; Note: Characteristics are based on non-imputed data. For categorical variables, percentages per category represent valid percentages. ^1^: NSES is a composite score based on (1) the average value of a house per 1,000 euros, (2) the percentage of owner-occupied houses, (3) the mean net disposable monthly income, and (4) the percentage of individuals aged 15-65 years receiving assistance benefits.

**Table S3.** Path coefficients from mediation analyses of frequency of fast-food consumption and amount of fat intake within the association between the fast-food environment and Body Mass Index among participants living in low neighbourhood socio-economic status areas with at least two healthy food outlets within 1km (*N* = *39,717*).

| **Food environment within 1 km** | **Paths** |  |  |  |  |  |  |  |  |  |
| --- | --- | --- | --- | --- | --- | --- | --- | --- | --- | --- |
| **Number of fast-food outlets** | **a1**  **OR (95% CI)** | **a2**  **B (95% CI)** | **b1**  **B (95% CI)** | **b2**  **B (95% CI)** | **c’**  **B (95% CI)** | **d**  **B (95% CI)** | **Mediated association frequency of fast-food consumption  B (95% CI)** | **Percentage mediated through frequency of fast-food consumption  B (95% CI)** | **Mediated association amount of fat intake B (95% CI)** | **Percentage mediated through amount of fat intake B (95% CI)** |
| 0 fast-food outlets | ref | ref | ref | ref | ref | ref | ref | ref | ref | ref |
| 2≥ fast-food outlets | 0.89 (0.67, 1.18) | **1.34 (0.27, 2.42)** | 0.09 (-0.03, 0.22) | **0.02 (0.01, 0.02)** | **0.74 (0.20, 1.27)** | **1.92 (1.71, 2.12)** | 0.00 (-0.01, 0.01) | -0.4% (-2.4%, 1.6%) | **0.02 (0.00, 0.04)** | **3.1% (0.6%, 5.5%)** |

Note: a1 = association between fast-food environment and frequency of fast-food consumption; a2 = association between fast-food environment and amount of fat intake; b1 = association between frequency of fast-food consumption and BMI; b2 = association between amount of fat intake and BMI; c’ = controlled direct association between fast-food environment and BMI; d = association between frequency of fast-food consumption and amount of fat intake. Bold values represent associations with p<0.05.

**Table S4.** Associations between the presence of fast-food outlets and Body Mass Index, stratified for the presence of healthy food outlets. Associations were adjusted for age, sex, partner status, highest level of completed education, weekly working hours, income, number of physical activity facilities within 1km, neighbourhood socio-economic status, household size, occupational prestige, and address density. Note: bold numbers represent associations with p<0.05.

| **Number of fast-food outlets within 1km** | **Number of healthy food outlets within 1km** | ***N*** | **BMI, B (95% CI)** |
| --- | --- | --- | --- |
| 0 fast-food outlets | 0 healthy food outlets | *23,758* | ref |
| 1 fast-food outlet | 0 healthy food outlets | *7,533* | **0.20 (0.07, 0.34)** |
| ≥2 fast-food outlets | 0 healthy food outlets | *4,325* | -0.08 (-0.27, 0.11) |
| 0 fast-food outlets | 1 healthy food outlet | *6,817* | ref |
| 1 fast-food outlet | 1 healthy food outlet | *8,318* | 0.15 (-0.05, 0.37) |
| ≥2 fast-food outlets | 1 healthy food outlet | *12,623* | **0.22 (0.02, 0.42)** |
| 0 fast-food outlets | ≥2 healthy food outlets | *2,832* | ref |
| 1 fast-food outlet | ≥2 healthy food outlets | *4,204* | -0.04 (-0.29, 0.22) |
| ≥2 fast-food outlets | ≥2 healthy food outlets | *79,106* | 0.11 (-0.11, 0.33) |

**Table S5.** Associations between the presence of fast-food outlets and Body Mass Index, stratified for both neighbourhood socio-economic status (NSES) and number of healthy food outlets within 1km. Associations were adjusted for age, sex, partner status, highest level of completed education, weekly working hours, income, number of physical activity facilities within 1km, household size, occupational prestige, and address density. Note: bold numbers represent associations with p<0.05.

| **Number of fast-food outlets within 1km** | **NSES^1^** | **Number of healthy food outlets within 1km** | ***N*** | **Body Mass Index,  B (95% CI)** |
| --- | --- | --- | --- | --- |
| 0 | Low | 0 | *1,050* | ref |
| 1 | Low | 0 | *724* | 0.41 (-0.10, 0.91) |
| ≥2 | Low | 0 | *1,610* | -0.18 (-0.67, 0.31) |
| 0 | Low | 1 | *547* | ref |
| 1 | Low | 1 | *1,891* | 0.38 (-0.19, 0.95) |
| ≥2 | Low | 1 | *3,840* | 0.54 (-0.03, 1.11) |
| 0 | Low | ≥2 | *395* | ref |
| 1 | Low | ≥2 | *1,253* | 0.37 (-0.25, 0.99) |
| ≥2 | Low | ≥2 | *38,069* | **0.75 (0.19, 1.31)** |
| 0 | Mid | 0 | *7,310* | ref |
| 1 | Mid | 0 | *2,901* | 0.07 (-0.17, 0.32) |
| ≥2 | Mid | 0 | *1,106* | -0.21 (-0.58, 0.16) |
| 0 | Mid | 1 | *3,015* | ref |
| 1 | Mid | 1 | *4,567* | 0.19 (-0.11, 0.49) |
| ≥2 | Mid | 1 | *4,577* | 0.06 (-0.26, 0.38) |
| 0 | Mid | ≥2 | *1,055* | ref |
| 1 | Mid | ≥2 | *1,741* | -0.21 (-0.62, 0.21) |
| ≥2 | Mid | ≥2 | *23,939* | -0.13 (-0.49, 0.22) |
| 0 | High | 0 | *15,398* | ref |
| 1 | High | 0 | *3,908* | **0.23 (0.06, 0.40)** |
| ≥2 | High | 0 | *1,609* | 0.07 (-0.20, 0.33) |
| 0 | High | 1 | *3,255* | ref |
| 1 | High | 1 | *1,860* | -0.01 (-0.33, 0.32) |
| ≥2 | High | 1 | *4,206* | 0.18 (-0.12, 0.48) |
| 0 | High | ≥2 | *1,382* | ref |
| 1 | High | ≥2 | *1,210* | 0.00 (-0.35, 0.35) |
| ≥2 | High | ≥2 | *17,098* | 0.05 (-0.24, 0.33) |

^1^: NSES is a composite score based on (1) the average value of a house per 1,000 euros, (2) the percentage of owner-occupied houses, (3) the mean net disposable monthly income, and (4) the percentage of individuals aged 15-65 years receiving assistance benefits.

**Table S6.** Associations between the presence fast-food outlets and waist-to-height ratio: for total study population, separately for low, middle, and high neighbourhood socio-economic status, and separately for participants with null, one, or at least two healthy food outlets within 1km. Associations were adjusted for age, sex, partner status, highest level of completed education, weekly working hours, income, number of physical activity facilities within 1km, household size, occupational prestige, and address density. Note: bold numbers represent associations with p<0.05.

| **Number of fast-food outlets within 1km** | **Subgroup^1^** | ***N*** | **Waist-to-height ratio, B (95% CI)** |
| --- | --- | --- | --- |
| 0 fast-food outlets | Total study population | *33,407* | ref |
| 1 fast-food outlet | Total study population | *20,055* | **0.002 (0.000, 0.003)** |
| ≥2 fast-food outlets | Total study population | *96,054* | 0.001 (0.000, 0.003) |
| 0 fast-food outlets | Low neighbourhood socio-economic status | *1,993* | ref |
| 1 fast-food outlet | Low neighbourhood socio-economic status | *3,868* | 0.004 (-0.001, 0.008) |
| ≥2 fast-food outlets | Low neighbourhood socio-economic status | *43,545* | **0.006 (0.001, 0.010)** |
| 0 fast-food outlets | Middle neighbourhood socio-economic status | *11,547* | ref |
| 1 fast-food outlet | Middle neighbourhood socio-economic status | *9,223* | 0.001 (-0.001, 0.004) |
| ≥2 fast-food outlets | Middle neighbourhood socio-economic status | *29,983* | -0.001 (-0.004, 0.002) |
| 0 fast-food outlets | High neighbourhood socio-economic status | *19,867* | ref |
| 1 fast-food outlet | High neighbourhood socio-economic status | *6,964* | 0.002 (-0.001, 0.004) |
| ≥2 fast-food outlets | High neighbourhood socio-economic status | *22,526* | 0.001 (-0.001, 0.003) |
| 0 fast-food outlets | Participants with 0 healthy food outlets within 1km | *23,758* | ref |
| 1 fast-food outlet | Participants with 0 healthy food outlets within 1km | *7,533* | **0.003 (0.001, 0.005)** |
| ≥2 fast-food outlets | Participants with 0 healthy food outlets within 1km | *4,325* | -0.002 (-0.005, 0.002) |
| 0 fast-food outlets | Participants with 1 healthy food outlet within 1km | *6,817* | ref |
| 1 fast-food outlet | Participants with 1 healthy food outlet within 1km | *8,318* | 0.003 (-0.003, 0.006) |
| ≥2 fast-food outlets | Participants with 1 healthy food outlet within 1km | *12,623* | **0.004 (0.003, 0.007)** |
| 0 fast-food outlets | Participants with ≥2 healthy food outlet within 1km | *2,832* | ref |
| 1 fast-food outlet | Participants with ≥2 healthy food outlet within 1km | *4,204* | 0.001 (-0.003, 0.005) |
| ≥2 fast-food outlets | Participants with ≥2 healthy food outlet within 1km | *79,106* | 0.003 (-0.001, 0.006) |

^1^: Neighbourhood socio-economic status is a composite score based on (1) the average value of a house per 1,000 euros, (2) the percentage of owner-occupied houses, (3) the mean net disposable monthly income, and (4) the percentage of individuals aged 15-65 years receiving assistance benefits.

**Table S7.** Associations between the presence fast-food outlets and waist-to-height ratio, stratified for both neighbourhood socio-economic status (NSES) and number of healthy food outlets within 1km. Associations were adjusted for age, sex, partner status, highest level of completed education, weekly working hours, income, number of physical activity facilities within 1km, household size, occupational prestige, and address density. Note: bold numbers represent associations with p<0.05.

| **Number of fast-food outlets within 1km** | **NSES^1^** | **Number of healthy food outlets within 1km** | ***N*** | **Waist-to-height ratio, B (95% CI)** |
| --- | --- | --- | --- | --- |
| 0 | Low | 0 | *1,050* | ref |
| 1 | Low | 0 | *724* | 0.007 (-0.001, 0.015) |
| ≥2 | Low | 0 | *1,610* | -0.002 (-0.010, 0.006) |
| 0 | Low | 1 | *547* | ref |
| 1 | Low | 1 | *1,891* | 0.005 (-0.004, 0.014) |
| ≥2 | Low | 1 | *3,840* | 0.007 (-0.002, 0.016) |
| 0 | Low | ≥2 | *395* | ref |
| 1 | Low | ≥2 | *1,253* | **0.010 (0.000, 0.020)** |
| ≥2 | Low | ≥2 | *38,069* | **0.017 (0.009, 0.027)** |
| 0 | Mid | 0 | *7,310* | ref |
| 1 | Mid | 0 | *2,901* | 0.000 (-0.004, 0.004) |
| ≥2 | Mid | 0 | *1,106* | -0.004 (-0.010, 0.002) |
| 0 | Mid | 1 | *3,015* | ref |
| 1 | Mid | 1 | *4,567* | **0.005 (0.000, 0.010)** |
| ≥2 | Mid | 1 | *4,577* | 0.003 (-0.003, 0.008) |
| 0 | Mid | ≥2 | *1,055* | ref |
| 1 | Mid | ≥2 | *1,741* | 0.001 (-0.006, 0.008) |
| ≥2 | Mid | ≥2 | *23,939* | 0.001 (-0.005, 0.007) |
| 0 | High | 0 | *15,398* | ref |
| 1 | High | 0 | *3,908* | **0.004 (0.001, 0.006)** |
| ≥2 | High | 0 | *1,609* | 0.001 (-0.004, 0.005) |
| 0 | High | 1 | *3,255* | ref |
| 1 | High | 1 | *1,860* | 0.000 (-0.005, 0.005) |
| ≥2 | High | 1 | *4,206* | 0.002 (-0.002, 0.007) |
| 0 | High | ≥2 | *1,382* | ref |
| 1 | High | ≥2 | *1,210* | -0.002 (-0.007, 0.004) |
| ≥2 | High | ≥2 | *17,098* | -0.002 (-0.007, 0.002) |

^1^: NSES is a composite score based on (1) the average value of a house per 1,000 euros, (2) the percentage of owner-occupied houses, (3) the mean net disposable monthly income, and (4) the percentage of individuals aged 15-65 years receiving assistance benefits.

**Table S8.** Path coefficients from the mediation analyses through frequency of fast-food consumption and amount of fat intake within the association between the fast-food environment and waist-to-height ratio among participants who lived in low neighbourhood socio-economic status areas and with at least two healthy food outlets within 1km (*N* = *39,717*).

| **Food environment within 1 km** | **Paths** |  |  |  |  |  |  |  |  |  |
| --- | --- | --- | --- | --- | --- | --- | --- | --- | --- | --- |
| **Number of fast-food outlets** | **a1**  **OR (95% CI)** | **a2**  **B (95% CI)** | **b1**  **B (95% CI)** | **b2**  **B (95% CI)** | **c’**  **B (95% CI)** | **d**  **B (95% CI)** | **Mediated association frequency of fast-food consumption** | **Percentage mediated through frequency of fast-food consumption  B (95% CI)** | **Mediated association amount of fat intake** | **Percentage mediated through amount of fat intake B (95% CI)** |
| 0 fast-food outlets | ref | ref | ref | ref | ref | ref | ref | ref | ref | ref |
| 2≥ fast-food outlets | 0.89 (0.67, 1.18) | **1.34 (0.27, 2.42)** | **0.003 (0.001, 0.005)** | **0.003 (0.002, 0.004)** | **0.018 (0.009, 0.026)** | **1.92 (1.71, 2.12)** | 0.000 (0.000, 0.000) | -0.3% (-1.8%, 1.0%) | **0.000 (0.000, 0.001)** | **2.0% (0.1%, 3.9%)** |

Note: a1 = association between fast-food environment and frequency of fast-food consumption; a2 = association between fast-food environment and amount of fat intake; b1 = association between frequency of fast-food consumption and BMI; b2 = association between amount of fat intake and BMI; c’ = controlled direct association between fast-food environment and BMI; d = association between frequency of fast-food consumption and amount of fat intake. Bold values represent associations with p<0.05.

**Table S9.** Sensitivity analysis on the association between fast-food outlet presence and BMI among participants recruited between 2012-2013 (*N* = *79,697)*.

| **Number of fast-food outlets within 1km** | **Subgroup^1^** | ***N*** | **Body Mass Index, B (95% CI)** |
| --- | --- | --- | --- |
| 0 fast-food outlets | Total study population | *17,376* | ref |
| 1 fast-food outlet | Total study population | *10,415* | 0.06 (-0.07, 0.19) |
| ≥2 fast-food outlets | Total study population | *51,877* | 0.10 (-0.04, 0.23) |
| 0 fast-food outlets | Low neighbourhood socio-economic status | *879* | ref |
| 1 fast-food outlet | Low neighbourhood socio-economic status | *2,025* | **0.42 (0.01, 0.82)** |
| ≥2 fast-food outlets | Low neighbourhood socio-economic status | *22,935* | **0.51 (0.13, 0.88)** |
| 0 fast-food outlets | Middle neighbourhood socio-economic status | *5,800* | ref |
| 1 fast-food outlet | Middle neighbourhood socio-economic status | *4,438* | -0.14 (-0.35, 0.07) |
| ≥2 fast-food outlets | Middle neighbourhood socio-economic status | *15,934* | -0.11 (-0.33, 0.11) |
| 0 fast-food outlets | High neighbourhood socio-economic status | *10,697* | ref |
| 1 fast-food outlet | High neighbourhood socio-economic status | *3,952* | 0.14 (-0.04, 0.31) |
| ≥2 fast-food outlets | High neighbourhood socio-economic status | *13,008* | 0.09 (-0.10, 0.29) |
| 0 fast-food outlets | 0 healthy food outlets within 1km | *12,697* | ref |
| 1 fast-food outlet | 0 healthy food outlets within 1km | *4,484* | 0.13 (-0.04, 0.30) |
| ≥2 fast-food outlets | 0 healthy food outlets within 1km | *2,593* | -0.03 (-0.27, 0.21) |
| 0 fast-food outlets | 1 healthy food outlet within 1km | *3,599* | ref |
| 1 fast-food outlet | 1 healthy food outlet within 1km | *3,738* | 0.05 (-0.22, 0.32) |
| ≥2 fast-food outlets | 1 healthy food outlet within 1km | *7,271* | **0.32 (0.06, 0.58)** |
| 0 fast-food outlets | ≥2 healthy food outlets within 1km | *1,080* | ref |
| 1 fast-food outlet | ≥2 healthy food outlets within 1km | *2,193* | 0.06 (-0.29, 0.42) |
| ≥2 fast-food outlets | ≥2 healthy food outlets within 1km | *42,013* | 0.11 (-0.19, 0.40) |
| 0 fast-food outlets | Low neighbourhood socio-economic status & 0 healthy food outlets within 1km | *524* | ref |
| 1 fast-food outlet | Low neighbourhood socio-economic status & 0 healthy food outlets within 1km | *417* | **0.71 (0.05, 1.36)** |
| ≥2 fast-food outlets | Low neighbourhood socio-economic status & 0 healthy food outlets within 1km | *892* | 0.10 (-0.50, 0.70) |
| 0 fast-food outlets | Low neighbourhood socio-economic status & 1 healthy food outlet within 1km | *208* | ref |
| 1 fast-food outlet | Low neighbourhood socio-economic status & 1 healthy food outlet within 1km | *901* | 0.46 (-0.34, 1.26) |
| ≥2 fast-food outlets | Low neighbourhood socio-economic status & 0 healthy food outlet within 1km | *1,887* | 0.77 (-0.02, 1.56) |
| 0 fast-food outlets | Low neighbourhood socio-economic status & ≥2 healthy food outlets within 1km | *147* | ref |
| 1 fast-food outlet | Low neighbourhood socio-economic status & ≥2 healthy food outlets within 1km | *707* | 0.50 (-0.35, 1.36) |
| ≥2 fast-food outlets | Low neighbourhood socio-economic status & ≥2 healthy food outlets within 1km | *20,156* | **0.83 (0.06, 1.60)** |
| 0 fast-food outlets | Middle neighbourhood socio-economic status & 0 healthy food outlets within 1km | *3,563* | ref |
| 1 fast-food outlet | Middle neighbourhood socio-economic status & 0 healthy food outlets within 1km | *1,547* | -0.11 (-0.42, 0.20) |
| ≥2 fast-food outlets | Middle neighbourhood socio-economic status & 0 healthy food outlets within 1km | *708* | -0.23 (-0.68, 0.22) |
| 0 fast-food outlets | Middle neighbourhood socio-economic status & 1 healthy food outlet within 1km | *1,823* | ref |
| 1 fast-food outlet | Middle neighbourhood socio-economic status & 1 healthy food outlet within 1km | *1,967* | -0.08 (-0.46, 0.31) |
| ≥2 fast-food outlets | Middle neighbourhood socio-economic status & 0 healthy food outlet within 1km | *2,845* | 0.06 (-0.33, 0.46) |
| 0 fast-food outlets | Middle neighbourhood socio-economic status & ≥2 healthy food outlets within 1km | *414* | ref |
| 1 fast-food outlet | Middle neighbourhood socio-economic status & ≥2 healthy food outlets within 1km | *924* | -0.18 (-0.76, 0.40) |
| ≥2 fast-food outlets | Middle neighbourhood socio-economic status & ≥2 healthy food outlets within 1km | *12,381* | -0.06 (-0.55, 0.43) |
| 0 fast-food outlets | High neighbourhood socio-economic status & 0 healthy food outlets within 1km | *8,610* | ref |
| 1 fast-food outlet | High neighbourhood socio-economic status & 0 healthy food outlets within 1km | *2,520* | 0.18 (-0.05, 0.40) |
| ≥2 fast-food outlets | High neighbourhood socio-economic status & 0 healthy food outlets within 1km | *993* | 0.13 (-0.21, 0.47) |
| 0 fast-food outlets | High neighbourhood socio-economic status & 1 healthy food outlet within 1km | *1,568* | ref |
| 1 fast-food outlet | High neighbourhood socio-economic status & 1 healthy food outlet within 1km | *870* | 0.06 (-0.36, 0.48) |
| ≥2 fast-food outlets | High neighbourhood socio-economic status & 1 healthy food outlet within 1km | *2,539* | **0.42 (0.04, 0.80)** |
| 0 fast-food outlets | High neighbourhood socio-economic status & ≥2 healthy food outlets within 1km | *519* | ref |
| 1 fast-food outlet | High neighbourhood socio-economic status & ≥2 healthy food outlets within 1km | *562* | 0.23 (-0.27, 0.73) |
| ≥2 fast-food outlets | High neighbourhood socio-economic status & ≥2 healthy food outlets within 1km | *9,476* | -0.05 (-0.45, 0.34) |

^1^: Neighbourhood socio-economic status is a composite score based on (1) the average value of a house per 1,000 euros, (2) the percentage of owner-occupied houses, (3) the mean net disposable monthly income, and (4) the percentage of individuals aged 15-65 years receiving assistance benefits.

**Table S10.** Repetition of the mediation analyses on participants recruited between 2012-2013 living in low NSES areas with at least two healthy food outlets within 1km (*N* = 21*,010)*.

| **Food environment within 1 km** | **Paths** |  |  |  |  |  |  |  |  |  |
| --- | --- | --- | --- | --- | --- | --- | --- | --- | --- | --- |
| **Number of fast-food outlets** | **a1**  **OR (95% CI)** | **a2**  **B (95% CI)** | **b1**  **B (95% CI)** | **b2**  **B (95% CI)** | **c’**  **B (95% CI)** | **d**  **B (95% CI)** | **Mediated association frequency of fast-food consumption  B (95% CI)** | **Percentage mediated through frequency of fast-food consumption  B (95% CI)** | **Mediated association amount of fat intake B (95% CI)** | **Percentage mediated through amount of fat intake B (95% CI)** |
| 0 fast-food outlets | ref | ref | ref | ref | ref | ref | ref | ref | ref | ref |
| 2≥ fast-food outlets | 0.78 (0.48, 1.28) | 0.64 (-0.14, 1.42) | 0.07 (-0.05, 0.20) | **0.02 (0.00, 0.04)** | **0.81 (0.37, 1.25)** | **1.47 (0.09, 1.85)** | -0.01 (-0.02, 0.01) | -0.5% (-5.4%, 4.3%) | 0.01 (-0.02, 0.04) | 1.5% (-2.8%, 5.9%) |

Note: a1 = association between fast-food environment and frequency of fast-food consumption; a2 = association between fast-food environment and amount of fat intake; b1 = association between frequency of fast-food consumption and BMI; b2 = association between amount of fat intake and BMI; c’ = controlled direct association between fast-food environment and BMI; d = association between frequency of fast-food consumption and amount of fat intake. Bold values represent associations with p<0.05.

**Table S11.** Results of the sensitivity analysis using frequency of fast-food consumption continuously instead of categorically in the mediation analysis regarding the association between the fast-food environment and BMI among participants from low neighbourhood socio-economic status with at least two healthy food outlets within 1km (*N* = *39,717*).

| **Food environment within 1 km** | **Paths** |  |  |  |  |  |  |  |  |  |
| --- | --- | --- | --- | --- | --- | --- | --- | --- | --- | --- |
| **Number of fast-food outlets** | **a1**  **OR (95% CI)** | **a2**  **B (95% CI)** | **b1**  **B (95% CI)** | **b2**  **B (95% CI)** | **c’**  **B (95% CI)** | **d**  **B (95% CI)** | **Mediated association frequency of fast-food consumption  B (95% CI)** | **Percentage mediated through frequency of fast-food consumption  B (95% CI)** | **Mediated association amount of fat intake**  **B (95% CI)** | **Percentage mediated through amount of fat intake B (95% CI)** |
| 0 fast-food outlets | ref | ref | ref | ref | ref | ref | ref | ref | ref | ref |
| 2≥ fast-food outlets | -0.04 (-0.13, 0.03) | **1.43 (0.36, 2.50)** | 0.02 (-0.07, 0.11) | **0.02 (0.01, 0.02)** | **0.74 (0.20, 1.27)** | **1.38 (1.22, 1.53)** | 0.00 (-0.01, 0.00) | -0.1% (-0.9%, 0.8%) | **0.02 (0.00, 0.04)** | **3.1% (0.6%, 5.5%)** |

Note: a1 = association between fast-food environment and frequency of fast-food consumption; a2 = association between fast-food environment and amount of fat intake; b1 = association between frequency of fast-food consumption and BMI; b2 = association between fat intake and BMI; c’ = controlled direct association between fast-food environment and BMI; d = association between frequency of fast-food consumption and fat intake. Bold values represent associations with p<0.05.
